# Abstract of Proceedings of the Buffalo Medical Association

**Published:** 1869-03

**Authors:** T. M. Johnson

**Affiliations:** Sec’y.


ART. III—*Abstract of Proceedings of the Buffalo Medical Association.*

TUESDAY EVENING, March 2d, 1869.

The meeting was called to order by the President. Members present—Drs. Lothrop, Miner, Little, White, Potter, Hauenstein, Wetmore, Cronyn, Samo and Johnson.

DR. MINER presented an eye, enucleated a few days since for encephaloid disease. The disease appeared about one year before its final removal, and was at first regarded by those who saw it as ptyrigum, and as such had several times been dissected from its attachment to the anterior border of the sclerotic, and nit. silver applied to prevent re-appearance; the last effort of this sort was made about three months since. Early return of the disease and rapid growth, together with constant pain, convinced his attending physician, Dr. Cornwell, and his son, Dr. Parmlee, that enucleation of the globe was the only operation which could offer any rational expectation of relief. The profession now unanimously assent to the early removal of the globe of the eye, when it is found to be the seat of malignant disease, but this operation is also called for in another and much larger class of diseases, and mainly on this account was the specimen presented, though of itself it had many points of interest. The tumor, now about one and a half inches in diameter, appeared not to have involved the interior of the globe, since vision remained until the cornea was covered by the overlapping of the fungus mass. The attachment appeared to be mainly to the sclerotic tunic of the eyeball, and did not extend far in it or to the conjunctiva, giving the mass the appearance of having a pedicle. It is now believed that malignant disease of the globe most frequently extends to the tissues in the orbit through the optic nerve; in this instance the nerve and its sheath were to all appearance perfectly healthy. The disease was completely removed so far as could be known by common inspection, and though it was done at far too late a period of its progress, it was undoubtedly proper and desirable.

Prof. White inquired of Dr. Miner his prognosis.

Dr. Miner replied: Encephaloid disease is actively malignant, and generally very rapidly progressive. I expect it will re-appear if life continue, either in the orbit or elsewhere. The removal was advised with the hope of at least temporary relief, without expectation of permanent cure.

Prof. White fully coincided with this view, and said his observation and experience fully sustained this prognosis, and the expectation of at least great temporary relief, which might continue some time.

Dr. Miner then proceeded to say, that the manner of operation now practiced by surgeons, was so unlike that adopted until within the last few years, that possibly a word in relation to it might not be wholly inappropriate.

Removal of the eye had formerly been justly regarded as one of the most painful and difficult operations in surgery, to be only adopted for removal of malignant disease, and as a last resort; whereas now, it could only be looked upon as one of the most simple and safe. The globe resting in a fibrous cup, (Tenon's capsule) could be removed with astonishing facility, if the anatomy of the parts was well understood. It now seemed most remarkable that surgeons had not earlier discovered the present simple method. If the conjunctiva is divided near the inner canthus, as in the operation for strabismus, and the tendon raised and divided in very similar manner, but much more freely; the curved scissors may now be passed back and divide the optic nerve, when it only remains to pass the same curved scissors around the globe, dividing the conjunctiva and the tendons of the oblique and recti muscles near their attachments to the sclerotic. The vessels divided are very small, and the hemorrhage is rarely troublesome—may always be easily controlled by cold water, ice or pressure.

*Enucleation of the globe* may be required not only in malignant disease, such as has been presented, but also much more frequently in all conditions of disease or injury causing what is called *sympathetic ophthalmia,* and to this point was it designed to invite particular attention. Patients, and even physicians, shrink from the suggestion of removing the eye when vision is wholly lost, and when the safety of the other eye is greatly endangered by delay. The fact of sympathetic inflammation is established beyond all doubt, and at the present time the necessity of removing an injured or diseased eye when such inflammation is induced by it in the opposite eye, is also as fully recognized by all who pay attention to ophthalmic surgery; however, the masses of the profession still require that their attention be directed to it, for the loss of vision often depends upon lack of appreciation of this fact in physicians, together with a natural dread of such operation on the part of patients. Fruitless efforts are long continued with the mistaken notion that this form of inflammation is in some degree amenable to ordinary remedies. The periods of increase and improvement so common in the disease mislead the unwary, and often the golden opportunity of saving vision in the healthy eye by timely operation, is lost, before the fatal error is discovered. The physician may be held justly responsible for loss of vision if he fails to appreciate the well established facts connected with this subject, and faithfully warn his patient of the danger.

It is well understood that irritations in the ciliary region cause *sympathetic ophthalmia;* that a variety of diseases of the cornea, iris, capsule of lens, etc., together with lodgment of foreign bodies in the globe produce this form of inflammation in the healthy eye, but the philosophy of this action or the structures through which it is conveyed are not so well understood. Formerly it was believed to be through the optic nerve; division and exsection of this nerve, however, does not interrupt the chain of sympathy, and it is now regarded as almost certain that the ciliary nerves are the media of this influence. Acting upon these views experimenters have divided the ciliary and optic nerves—have exsected the optic nerve, and in various ways attempted to interrupt the function of the ciliary, but as yet no very important results have been gained; these attempts have been made, always however, reserving the established and certain operation of enucleation as a final resort.

With the same view many other operations upon the globe have been long practiced: Puncture of the globe, thus allowing the escape of the contents; excising the corneal or anterior portion, and, later, dividing the globe back of the ciliary region, after it was discovered that it was probably the site of sympathetic irritation, have all been practiced. A desire to save a portion of the globe of the eye for the purpose of better adjustment and motion of artificial eye has been almost universal, and some modes of operation worthy of mention have been proposed. The tunics of the eyeball were drawn together by sutures, but it appeared that the presence of suture in the sclerotic kept up the irritation, and were objectionable. Prof. Knapp of Heidelburg, has proposed suture of the conjunctiva alone to cover the stump after dividing the globe back of the ciliary region, and this may prove advantageous in some cases. The probabilities are, that really the best method is enucleation of the entire globe, since it is followed by more rapid recovery, is more certain in its results, produces less local disturbance, and moreover affords nearly or quite as satisfactory support for an artificial eye. When the globe is enucleated, the artificial eye can be early worn, rests upon the fibrous capsule without irritation, is moved in the various directions by the muscles which after separation from the globe form attachments to the fibrous capsule, and thus all sources of sympathetic irritation are at once and certainly removed. The great argument for retaining a part of the globe, is briefly answered by Græfe, who says: "Enucleation of the globe affords nearly as good, and in many cases a much better support for the artificial eye."

It may be necessary to remove the globe of the eye in all injuries causing iro-choroideitis; iritis from immovable causes, as when it is involved in a wound; disease of choroid or cornea, or of the vitrious body. Above all, the lodgment of a foreign body frequently necessitates the enucleation of the globe. Disease of the capsule of the lens, choroideitis from reclination or depression of the lens, separation of the retina by effusions; tumors, and all other diseases causing *sympathetic ophthalmia*, require removal of the globe.

The early symptoms of sympathetic inflammation are of importance, and should be carefully observed by the medical attendant, since upon it may depend the loss or preservation of vision. Intolerance of light, lachrymation, dimness of vision, partial loss of accommodation, contraction of the field of vision, pain, and tenderness over the ciliary region, immobility of the pupil, effusions upon the iris, advance of the iris towards the cornea as from pressure, are symptoms which may be present in some degree, at least, while the eye is yet capable of partial or complete recovery if the diseased eye is removed. These symptoms being aggravated and others added, such as complete blindness, closure of pupil from effusion of lymph, separation of the retina and other intra ocular changes and no hope of recovery can be indulged, vision is hopelessly lost and operative interference is no longer advisable with any view of restoration.

Dr. White suggested that Dr. Miner relate the case of a patient where he had invited him to assist in enucleation of the globe, in which was lodged a foreign body, since it would illustrate in some respects the subject he had presented.

Dr. Miner remarked that he could not perhaps relate the details of the case, though it would illustrate many points in connection with sympathetic ophthalmia. Capt. G. was injured by the lodgment of a piece of steel in the globe of the eye sometime in June last. The steel entered through the cornea, making an extensive incision. He was near Detroit, and was there treated by a most excellent ophthalmic surgeon, Dr. Noyes, who made repeated unsuccessful attempts to discover and remove the foreign body.

Partially recovering, he returned to his home in Buffalo and received advice from Prof. White. The symptoms of sympathetic ophthalmia were mild, and at times would seem almost wholly absent, again to re-appear and become as troublesome as ever. Dimness of vision, photophobia and lachrymation were the more prominent symptoms. The globe of the injured eye was shrunken, and the cornea, or what of it remained, was opaque. At Prof. White's suggestion he was consulted as to the necessity of removing the globe of the injured eye. Wholly agreeing with the proposition, he urged the importance of an immediate operation, which was gladly assented to. The steel was found in the center of the contracted globe, which now had none of the appearances and contained nothing of the humors of the natural eye; the tunics of the globe had contracted down upon the steel, now surrounded by lymph. The steel was about half an inch in length, a fourth inch in breadth, and a line in thickness. The wound healed kindly, all the symptoms of irritation in the healthy eye soon disappeared, and the patient is now wearing an artificial eye with comfort and almost perfect restoration of former appearance.

Similar illustrations were a frequent, very frequent occurrence, and the only remarkable feature of this case was the mildness of the symptoms in the sound eye; more commonly, violent sympathetic inflammation would sooner and more urgently demand removal.

Dr. White said, I arise to bear testimony to the propriety of this operation as a means of saving the sound eye. In the case last mentioned by Dr. Miner, I had delayed the operation for considerable time, hoping that the foreign body had escaped. Notwithstanding, the patient had suffered so much in the sound eye from sympathetic irritation that I advised its removal. The operation disclosed the fact that the morbid condition was kept up by a spicula of metal half an inch in length by two lines in width, which was now discovered. The stump recovered without any untoward symptoms, and has had an artificial eye fitted to it which performs its new functions satisfactorily. The remaining eye has completely recovered since the extirpation of the diseased one.

When in London in 1866, I found that both Bowman and Critchett in the Royal Ophthalmic Hospital, urged the importance of removal of the unsound eye for the purpose of saving the sound one when sympathetically affected. Although this operation had a London origin, being first made by the first gentlemen named, I believe it has been now adopted both in Europe and America, and I think has received too little attention from the profession, and am therefore happy that the subject has been brought up here by Dr. Miner.

Dr. Lothrop said that the case was interesting, and as far as he knew, rare. He had never seen one like it, though he had often seen cases of encephaloid disease of the globe and orbit. Malignant disease arising from the external surface of the eye, etc., thought must be very infrequent, and he believed that was the opinion of authors. There is sometimes seen a fleshy fungus mass growing from the surface of the eye, bleeding easily, and having much the appearance of malignant disease, but not truly malignant. The last arose more often from the optic nerves, or its expansion and gradually filling the globe finally pushed outwards, breaking through near the cornea and rapidly growing. In the case related this could not have been the process, as sight was retained for sometime, even while the external growth was enlarging. The specimen now presented he thought, without doubt, malignant, and though apparently thoroughly removed, almost sure to return.

He could fully concur with Dr. Miner, in remarks concerning the modern method for removal of the globe. The operation as now practiced had ceased to be, as formerly styled, a truly formidable operation. Earlier in life he had witnessed the formidable operation of removing the whole contents of the orbit, and also the incision for evacuating the contents of the globe. Neither of them were pleasant to see or do, but the first was especially to be dreaded. Such being the case, there was often hesitation in removing the globe in those cases in which the sound eye was endangered from sympathetic ophthalmia, and consequently in many cases an injury to one eye eventuated in blindness. The modern operation was not one difficult to perform, and there should be no delay in practicing it. He was glad of an opportunity of saying something in confirmation of the recommendation of Dr. Miner, viz. : an early removal of the injured globe upon the earliest appearance of the unmistakable signs of sympathetic affection of the sound eye, otherwise it will be likely to go on to entire and hopeless blindness.

Hesitation might be felt, if the operation were not simple and effectual.

Dr. Lothrop thought that the reason of the reluctance to remove the whole globe, arose from the belief that when the globe was removed the stump remaining upon which to place an artificial eye was imperfect and inferior to that formed by leaving a portion of the globe. This is now known to be partly an error, besides that there is greater liability to irritation and increased suppuration. Although this liability was recognized, still it was believed that when only the muscles and fibrous capsule exterior to the globe were left, the artificial eye was less comely and less easily controlled. It appears, however, that the stump left after removal of the globe serves well to control the motions of the artificial eye, and is nearly if not quite as useful as when part of the globe is left. He could not, however, but think that leaving out of consideration the increased irritation and suppuration which results, and regarding only comeliness, there was a decided advantage in leaving a portion of the globe. When the whole is removed the artificial eye looks slightly smaller and sunken.

Dr. Hauenstein objected to the report of his case of Spurious Melanosis, reported at the January meeting as published in the *Buffalo Medical and Surgical Journal* for February. Dr. Miner made motion, which was carried, that he be invited to report his case in full as correction upon that already published.

Dr. Hauenstein then said: Mr. President, with the permission of this Association I take the liberty to present the following corrected statement of the case of Spurious Melanosis, as published under "Abstract of Proceedings" of this Association in the February number of the *Buffalo Medical and Surgical Journal:*

In view of the rapid increase of manufacturing establishments in our city, and more particularly of those which expose persons therein engaged as workmen to the inhalation of carbonaceous matter, and since it is quite probable that spurious melanosis, so called, is more frequently the cause of death than we are aware of, and deserves to be studied; therefore the case in question is one of interest.

In reporting this case, it is with the view only to call the attention of the medical profession in this city to the subject, not being able, I regret to say, to throw any new light upon its ætiology, symptoms or treatment.

The subject of the case was a partner in a foundry business, and his specialty was that of a moulder. He was a hard worker, and up to November 30th, the day previous to my first visit, was daily engaged in that kind of work, and considered himself healthy with the exception of a slight cough, which had troubled him for the last three weeks, and which was attended with a little expectoration of unaltered mucus, (judging by his description,) and considered by him a catarrhal affection brought on by a "cold."

The case, after my first visit, was diagnosed and treated as a case of pleuritis, (never as pneumonia,) in which opinion I was sustained by Drs. Lothrop and Rochester, and for which opinion and view of the case there was ample reason as the *post mortem* examination has shown. The only symptoms that excited apprehension, in his chest, of more serious trouble than pleuritis, was a few days before the case went out of my hands, that after a lengthy paroxysm of coughing, a strong fetor in his breath was perceptible, but which was not imparted to the expectoration, which, even at this late period, from the thirteenth to the fifteenth day, was still a clear and transparent mucus.

The patient then came into the hands of Dr. Tobie, who informs me that four or five days after his first visit, the patient expectorated two or three pints of black, very fetid liquid, attended with such a degree of prostration as to threaten speedy dissolution; after which the patient rallied again and gave renewed hope of recovery, but eight or ten days later another attack of the same character put an end to his existence.

The friends of the patient, attributing his death to a severe blow upon the middle third of the sternum, which he received about three months before his death, the effects of which were severely felt at the time, requested coroner Dr. Diehl to hold an inquest, and in the presence of Drs. Tobie, Chase and myself, a *post mortem* examination was made December 28th, about twelve hours after death.

In describing the morbid appearances found in the lungs, I can make use, almost verbatim, of the language of Dr. C. G. Gregory of Edinburgh, who first described the *post mortem* appearances observed in the lungs of a patient who died of spurious melanosis, an account of which is contained in an article on the subject in the *Cyclopædia of Practical Medicine.* Both lungs presented one uniform black carbonaceous color, pervading every part of their substance. The lower lobe of the left lung was much disorganized, and exhibited in its lower half a large, irregular cavity, containing a large quantity of black liquid, like ink; several smaller cavities were observed in the same lobe. The upper lobe of the left lung, although presenting the same uniform black color, was quite pervious to air, and was in fact the only portion of the lungs that could, apparently, perform its function. The entire right lung was condensed and loaded with black serum. The serum when pressed, was of the same black color as the lungs. No cavities were found in the right lung. The lungs were everywhere adherent, the left more so than the right; some of the adhesions had considerable firmness. The pericardium contained from seven to eight ounces of *clear, transparent* serum. The heart seemed to be healthy. The liver was somewhat enlarged; kidneys and spleen healthy.

Dr. Miner offered the following resolution, which was laid on the table until the next meeting:

*Resolved,* That the fee for examination for life insurance shall be five dollars when made in the offices of physicians, and two dollars additional when the examination is made outside the office.

Dr. Miner also introduced the following with the request that it be laid on the table until the next meeting:

*Resolved,* That it shall be regarded as a breach of confidence for druggists to renew prescriptions upon which is written, *not to be renewed,* or any other sign or device by which it can be known that such renewal is against the wishes of the prescriber.

Adjourned. T. M. Johnson, Sec'y.

Acupressure at the New York Hospital.—Since the first of December acupressure has been employed at this hospital, in two amputations at the shoulder joint, in two of the thigh, and in one at the knee-joint, with complete prevention of hemorrhage in every case. All the cases but one, which died of pyæmia, either have recovered or are in a fair way to do so.—*Medical Record.*

